# Melasma Revisited: National Survey Reveals How Dermatologists Diagnose and Treat This Complex Skin Condition

**DOI:** 10.1111/jocd.16630

**Published:** 2024-10-15

**Authors:** Pelin Hizli, Fatma Arzu Kiliç, Seyma İçöz Aytaç

**Affiliations:** ^1^ Department of Dermatology, Faculty of Medicine Balikesir University Balikesir Turkiye

**Keywords:** chemical peeling, dermatologist, laser, melasma, tranexamic acid

## Abstract

**Background:**

Melasma is a chronic condition characterized by dark patches on the facial skin. It has a known female gender dominancy, and women usually think of melasma as being a serious cosmetic problem. Treatment of melasma can be seriously challenging, thus, dermatologists may employ different approaches for melasma. This study aimed to investigate how dermatologists diagnose and treat patients with melasma and to present the general characteristics of patients with melasma.

**Methods:**

A survey was conducted using Google Forms targeting dermatologists in Turkiye. A total of 199 dermatologists (142 females/57 males) participated in the study.

**Results:**

Most of the participants (52.3%) were residents. Majority gender of the patients was female and most common age range of patients with melasma was 30–40 years. Mixed melasma was the most common type (57.4%). Malar region was the most frequent location (58.6%). Wood's lamp examination was used by 25.6% of the participants. Topical treatment was the first‐line choice for melasma therapy (95%), with Kligman's formula being the most used topical agent (69.8%). Oral therapy was not used by 70.8% of the participants. Tranexamic acid was the first choice for oral treatment (23.4%). Only 24.9% of the dermatologists used laser/light therapy, and Q‐switched Nd‐YAG laser was the most preferred device (58%). The most common recurrence rate was 41%–60% (45.9%).

**Conclusions:**

The findings of the current study investigating the melasma management in Turkiye revealed a female predominance and peak prevalence in the 30–40 years age group. Kligman's formula is the preferred topical treatment, whereas oral tranexamic acid remains underutilized. Recurrence rates are high, highlighting the need for preventative strategies. This study emphasizes the importance of personalized approaches and ongoing research for effective melasma management.

## 1 Introduction

Melasma is a chronic acquired hyper‐melanosis disorder characterized by the presence of symmetrical brown‐gray macules primarily affecting sun‐exposed areas of the face and neck. Individuals with Fitzpatrick skin phototypes IV–VI are more susceptible [1]. Commonly affected areas include the cheeks, forehead, upper lip, nose, and chin [2]. A single macule or multiple macules can be observed in patients with melasma. Macules can vary in morphology, appearing confetti‐like, guttate, linear, or confluent. Development is typically gradual, occurring over weeks to years, usually fades in the winter, and becomes evident in the summer [3]. Melasma can be observed clinically in three patterns depending on the location of the macules. Centro‐facial pattern is the most common pattern, affecting the forehead, cheeks, upper lip, and chin. The lateral cheeks are affected in the malar pattern and the mandibular pattern is the least common form affecting the lower jawline. Fifty to eighty percent of the patients have centro‐facial type melasma [4].

Melasma can be categorized as four groups on the basis of Wood light examination: epidermal, dermal, mixed, and indeterminate form. The epidermal form is lighter brown and there is an increase in contrast during Wood examination. The dermal form is brown, bluish gray in color. There is no contrast increase in wood light examination, but in the mixed type, there is a dark brown appearance, the contrast increase occurs in a certain area [5].

Melasma has a known female gender dominancy, and women usually think of melasma as being a serious cosmetic problem. The presence of miscellaneous clinical patterns and features of the disease might bring about different diagnose and treatment and follow‐up approaches among dermatologists, including a quite difficult and long treatment process. Based on this perspective, this study aimed to evaluate the effectiveness of various diagnostic and therapeutic approaches for melasma in a Turkish population. Specifically, it aimed to characterize patients with melasma, assess diagnostic methods, treatment options and recurrence rates, and identify the factors influencing treatment success, to detect the treatment options most associated with recurrence and reasons of the patients for breaking the treatment up.

## 2 Materials and Methods

This research was reviewed and approved by the institutional ethical committee (IEC) of Balikesir University (registration number: 2023/15) and all procedures of this study were performed in accordance with the Helsinki Declaration. Informed consent was obtained from all participants. We conducted a cross‐sectional survey of Turkish dermatologists to assess their diagnostic and treatment approaches for melasma and to gather national data on patients with melasma encountered by these physicians. A random sample of dermatologists was drawn from the national database of Turkiye ensuring representation from all geographical regions and seniority levels (research assistants to professors). According to the Ministry of Health records, there exist 1077 dermatologists working in Turkiye. Although the total number of dermatologists is difficult to ascertain because of the inclusion of academics and residents, current estimates place the figure between 2000 and 3000. All participating dermatologists were invited via multiple methods (e.g., email and phone calls). A self‐administered questionnaire was developed using Google Forms and consisted of validated questions addressing dermatologist demographics, melasma diagnosis and treatment practices, and patient characteristics. The survey questions were developed by experts in the field with a thorough understanding of melasma's medical characteristics, following a comprehensive literature review. The questions were then pilot‐tested in a small group of dermatologists to ensure their validity and reliability. The survey achieved a response rate of 89.5%, with 199 physicians providing valid data among the 220 contacted. Descriptive statistics were employed to analyze the survey data. Results were presented as frequencies and percentages.

## 3 Results

In total, 199 physicians (142 females and 57 males) participated in the study. The features of the participant physicians are presented in Table 1. The majority of the participants were in the 24–30 years age group (90 physicians—45.2%) and had a work experience of 1–5 years (53.3%) in dermatology. Dermatologists working in university hospitals (68 dermatologists—34.3%) mostly participated in the study. Most of the participants were residents (104 physicians—52.3%), followed by specialists (59 physicians—29.6%), and most of the participants were working in the Marmara region of Turkiye (57 physicians—28.8%), followed by mid‐Anatolian region (48 physicians—24.2%).

**TABLE 1:** Features of the participant physicians.

		*N*	%
Age	24–30	90	45.2
	30–40	46	23.1
	40–50	38	19.1
	50–60	24	12.1
	60–70	1	0.5
Gender	Female	142	71.4
	Male	57	28.6
Work experience in dermatology	1–5	105	53.3
	6–10	20	10.2
	11–15	17	8.6
	16–20	17	8.6
	21–25	16	8.1
	26–30	12	6.1
	31–35	8	4.1
	36–40	2	1
Current institution/type of healthcare cent	University	68	34.3
	Training and research hospital	39	19.7
	City hospital	30	15.2
	Public hospital	17	8.6
	Private hospital	14	7.1
	Private practice	30	15.2
Position	Resident	104	52.3
	Specialist	59	29.6
	Associate professor	11	5.5
	Assistant professor	8	4
	Professor	17	8.5
Region	Marmara	57	28.8
	Aegean	22	11.1
	Mediterranean	22	11.1
	Black Sea	30	15.2
	Mid‐Anatolian	48	24.2
	Southeastern Anatolian	13	6.6
	Eastern Anatolian	6	3
Number of patients admitted to outpatient clinic daily	0–15	24	12.1
	16–30	29	14.6
	31–45	39	19.7
	46–60	35	17.7
	61–75	40	20.2
	75–90	18	9.1
	> 90	13	6.6
Number of patients with melasma seen monthly	0–15	89	44.7
	16–30	54	27.1
	31–45	25	12.6
	46–60	10	5
	61–75	9	4.5
	75–90	6	3
	> 90	6	3

Table 2 presents the features of the patients with melasma admitted by the participant physicians. Majority gender of the patients was female according to 198 (99.5%) physicians and most common age range of patients with melasma was 30–40 years according to 163 (81.9%) physicians. The most common type of melasma was reported as mixt by 113 (57.4%) physicians, the most common melasma location was reported as malar region by 116 (58.6%) physicians, and the most common season of melasma were reported as summer by 77 (38.7%) physicians.

**TABLE 2:** Features of the patients with melasma.

Majority gender of patients with melasma	Mostly female	198	99.5
	Mostly male	1	0.5
	Female male equal	0	0
Most common age range of patients with melasma	< 18	1	0.5
	18–25	0	0
	25–30	3	1.5
	30–40	163	81.9
	40–50	31	15.6
	50–60	1	0.5
	60–70	0	0
Most common type of melasma	Epidermal	75	38.1
	Dermal	9	4.6
	Mixt	113	57.4
Most common type of melasma location	Malar	116	58.6
	Centro‐facial	73	36.9
	Mandibular	1	0.5
	Extra‐facial	1	0.5
	Upper lip	7	3.5
The most common season that patients with melasma apply?	Summer	77	38.7
	Autumn	33	16.6
	Winter	13	6.5
	Spring	27	13.6
	No seasonal variation	49	24.6

The distribution of the responses of the participants to the questions focusing on the general aspect to the diagnosis and treatment of melasma was presented in Table 3. The rate of wood lamp examination among physicians was 25.6% (51 physicians). The most common risk factors of melasma reported by physicians were sun exposure (190 physicians—95.5%), pregnancy history (189 physicians—95%), and hormonal therapy (152 physicians—76.4%). Ninety‐seven physicians (48.7%) preferred chemical (organic) sunscreens; 129 physicians (65.5%) selected sunscreens with SPF over 50. Definite recommendation rate of physical sun protection was 64.3% (128) among all physicians. One hundred and sixty‐five (83.3%) physicians reported that they preferred to manage melasma in autumn–winter season.

**TABLE 3:** Distribution of the responses of the participants to the questions focusing on the general aspect to the diagnosis and treatment of melasma.

		*N*	%
Do you perform wood lamp examinations on patients with melasma?	Yes. I use it on every single patient	51	25.6
	No	37	18.6
	I don't have wood lamp	6	3
	If I have a chance. I will	105	52.8
Which of the risk factors do you encounter in patients with melasma?	Family history	72	36.2
	Sun exposure	190	95.5
	Pregnancy history	189	95
	Hormone therapy	152	76.4
	Job	98	49.2
	Oral contraceptive use	150	75.4
	Phototoxic drug use	110	55.3
	Concomitant thyroid disease	70	35.2
	I don't question	2	1
What is your preference in sunscreens?	Chemical (organic) sunscreens	97	48.7
	Physical (inorganic) sunscreens	52	26.1
	No idea	6	3
	I don't have a special preference	44	22.1
What is your SPF selection in sunscreen preference?	SPF15	1	0.5
	SPF30	5	2.5
	SPF50	62	31.5
	SPF50+	129	65.5
	No idea	0	0
	I don't have a special preference	0	0
Do you prefer tinted sunscreens primarily?	Yes	128	64.3
	No	38	18.1
	No idea	3	1.5
	I don't have a special preference	32	16.1
Do you recommend physical sun protection methods in addition to sunscreens to patients with melasma?	I definitely recommend it to every patient	138	69.7
	I recommend it if I find the time	44	22.2
	As a result of my clinical evaluation I recommend it to some patients	14	7.1
	I would never recommend	2	1
Do you have a seasonal preference for melasma treatment?	Autumn–winter	165	83.3
	Spring–summer	2	1
	I don't have a special preference	31	15.7

Table S1 shows the distribution of the responses of the participants to the questions focusing on the details of the treatment of melasma. The vast majority of the participant physicians (189 physicians—95%) preferred topical preparations as the first step in the treatment. The most recommended topical agents primarily were Kligman's formula (139 physicians—69.8%), hydroquinone (116 physicians—58.3%), and azelaic acid (113 physicians—56.8%). Chemical peels were usually not under the interest of the majority of the physicians (only 47 physicians—23.6%); however, glycolic acid was the most used chemical peeling agent (24 out of 47 physicians—51.1%). Sixty‐one physicians (31.1%) reported that they suggest dermo‐cosmetic product to the 81%–100% of their patients with melasma. The most preferred dermo‐cosmetic products were vitamin C (111 physicians—58.1%), niacinamide (90 physicians—47.1%), and arbutin (89 physicians—46.6%). The most common continuation rate of topical treatment was 41%–60%, reported by 61 (30.7%) of the physicians. The most common reason for the discontinuation of topical treatment was patient's incompatibility, reported by 137 (69.5%) of the physicians. Out of 192 physicians, 136 (70.8%) reported that they did not use oral therapy; however, tranexamic acid was most common first choice of oral treatment among the 192 physicians responded this question (45 physicians—23.4%). The reason for not using tranexamic acid was having no experience of tranexamic acid treatment (108 out of 154 physicians—70.1%). Only 49 (24.9%) physicians had the opportunity of laser/light therapy, and the most common preferred laser device was Q‐switched Nd‐YAG laser (29 physicians—58%). The most common recurrence rate was 41%–60%, reported by 83 (45.9%) of the participant physicians. One‐hundred and fifteen physicians (59.3%) reported that they provided maintenance therapy to the patients with melasma.

## 4 Discussion

Melasma is a disease with different approaches, and difficulties in the diagnosis and treatment. This study investigated the diverse approaches of dermatologists in Turkiye employ to diagnose and treat melasma, offering valuable insights into both patient characteristics and physician practices. By surveying the physicians from all seniority levels (research assistants, residents, and professors), we captured a broad spectrum of perspectives within the field. Drawing from a pool of approximately 2000–3000 dermatologists identified in the national database, 220 were recruited for this study. With usable data from 199 participants, the study achieved a participation rate of approximately 10%. According to our results, most of the participant physicians were in the 24–30 years age group and research assistants working at university hospitals. In Turkiye, patients with melasma were admitted by physicians from various regions and with miscellaneous titles, thus approaches may differ among every single physician or institute.

Our findings align with the current literature, demonstrating a female predominance (99.5%) and a peak prevalence in the 30–40 years age group [6, 7, 8, 9]. Thus, we can state that melasma has a female predominancy, and it is commonly seen in the fourth decade. Interestingly, although previous reports identified centro‐facial melasma as the most common type (55.44%) [6], our study revealed malar region melasma as the most frequently reported (58.6%). Additionally, mixed type melasma based on pigment location presented as the most prevalent (57.4%), contrasting with Achar and Rathi's [6] findings of dermal melasma being the most common (54.48%). These discrepancies highlight potential regional variations in melasma presentation. Moreover, although our study did not include certain data on regional discrepancies of melasma, it might have a regional variation. The higher melasma occurrence in Sanliurfa (Southeastern Anatolian region) was explained by predisposing factors like the higher fertility rates and sun exposure, in the report by Demirkan, Gündüz, and Sayan [10].

Although melasma remains a challenging and sometimes frustrating condition for both patients and physicians, advancements in therapeutic options offer promise. Kligman's formula, a well‐established combination of hydroquinone, tretinoin, and corticosteroid, emerged as the most preferred treatment modality according to the prior literature [5]. Consistently, Kligman's formula was the most frequently preferred treatment agent among the participant physicians in our study. However, a significant gap exists in the utilization of oral tranexamic acid, a potentially effective and well‐tolerated option [11, 12, 13]. The most common reason cited for its underutilization was the lack of experience among participating physicians in our study (70.1%). This finding underscores the need for increased awareness and education regarding this promising treatment avenue.

Among the participants of our study, only 24.9% of participants reported access to laser/light therapy, with Q‐switched Nd‐YAG lasers being the preferred device, consistent with current literature [14]. Our clinical protocol for patients with melasma involves the initial implementation of topical therapies. Kligman's formula is our primary topical regimen, and for patients exhibiting resistance to this treatment, we often adjunctively employ tranexamic acid. Furthermore, laser‐based procedures are considered as an additional therapeutic option within our treatment algorithm.

In this study, the participants were also questioned about the recurrence rate of melasma. The most frequently reported recurrence rate was 41%–60%, suggesting a significant challenge in achieving long‐term remission, reported by 45.9% of the participant physicians. Although definitive data on recurrence rates remain elusive because of limited long‐term follow‐up studies [15], our findings support the notion that melasma often recurs, warranting further investigation into preventative and maintenance strategies.

Although this study contributes meaningfully to the existing knowledge base, acknowledging its limitations is crucial. As a survey‐based study, the data may not provide definitive rates, highlighting the need for future multicenter studies incorporating patients directly. However, such surveys remain invaluable in revealing the general approaches employed by physicians toward specific patient groups.

## 5 Conclusion

This study investigates melasma diagnosis and treatment practices in Turkey. Findings reveal a female predominance and peak prevalence in the 30–40 years age group. Kligman's formula is the most preferred topical treatment, whereas oral tranexamic acid remains underutilized. Recurrence rates are high, highlighting the need for preventative strategies. This study emphasizes the importance of personalized approaches and ongoing research for effective melasma management. Moreover, although this study sheds light on the current landscape of melasma management in Turkiye, it underscores the urgent need for further research to develop more effective and innovative treatment approaches that can address the persistent challenges associated with this condition.

## Author Contributions

**Pelin Hizli:** conceptualization, methodology, software, formal analysis, investigation, writing – original draft, writing – review & editing. **Fatma Arzu Kiliç:** validation, writing – review & editing, supervision, project administration. **Seyma İçöz Aytaç:** formal analysis, investigation, resources, data curation, writing – review & editing, visualization.

## Consent

All participants have participated in the study providing their own written informed consent.

## Conflicts of Interest

The authors declare no conflicts of interest.

## Supporting information


Table S1

## Data Availability

The data that support the findings of this study are available from the corresponding author upon reasonable request.
