# Supplementary material for: Melasma Revisited: National Survey Reveals How Dermatologists Diagnose and Treat This Complex Skin Condition
Source: J Cosmet Dermatol. 2024 Oct 15;24(1):e16630. doi: 10.1111/jocd.16630 (PMC11743328; doi:10.1111/jocd.16630)
Supplement: Supplementary file 1 — Table S1 [file JOCD-24-e16630-s001.docx]

**Table S1**. The distribution of the responses of the participants to the questions focusing on the details of the treatment of melasma.

|  |  | N | % |
| --- | --- | --- | --- |
| What is your preference as the first step in the treatment? | Topical preparations | 189 | 95 |
|  | Oral preparations (Tranexamic acid, Polypodium leucotomos extract etc) | 0 | 0 |
|  | Chemical peelings | 2 | 1 |
|  | Mesotherapy | 1 | 0.5 |
|  | Platelet rich plasma | 1 | 0.5 |
|  | Microneedling | 1 | 0.5 |
|  | Radiofrequency microneedling | 0 | 0 |
|  | Dermabrasion | 0 | 0 |
|  | Lasers and light therapies | 5 | 2.5 |
| Which of the topical agents do you recommend primarily? | Hydroquinone | 116 | 58.3 |
|  | Azelaic acid | 113 | 56.8 |
|  | Topical steroids | 64 | 32.2 |
|  | Retinoids | 104 | 52.3 |
|  | Kojic acid | 11 | 5.5 |
|  | Niacinamide | 27 | 13.6 |
|  | Resveratrol | 3 | 1.5 |
|  | Cysteamine | 2 | 1 |
|  | Tranexamic acid | 27 | 13.6 |
|  | Kligman formula | 139 | 69.8 |
|  | Non-Kligman topical formulation | 2 | 1 |
| Do you use chemical peels? | Yes | 47 | 23.6 |
|  | No | 152 | 76.4 |
| Which chemical peeling agents do you prefer most? | Glycolic acid | 24 | 51.1 |
|  | Trichloroacetic acid | 16 | 34 |
|  | Pyruvic acid | 2 | 4.3 |
|  | Mandelic acid | 9 | 19.1 |
|  | Phytic acid | 2 | 4.3 |
|  | Jessner's solution | 8 | 17 |
| How many percent of your patients with melasma do you suggest dermo-cosmetic products in addition to the treatment? | 0-20 | 31 | 15.8 |
|  | 21-40 | 34 | 17.3 |
|  | 41-60 | 44 | 22.4 |
|  | 61-80 | 26 | 13.3 |
|  | 81-100 | 61 | 31.1 |
|  |  |  |  |
| If you add dermo-cosmetics what is/are your first choice(s)? | Niacinamide | 90 | 47.1 |
|  | Arbutin | 89 | 46.6 |
|  | Retinoids | 86 | 45 |
|  | Vitamin C | 111 | 58.1 |
|  | Kojic acid | 34 | 17.8 |
|  | I don’t add | 7 | 3.7 |
|  | Combination | 7 | 3.7 |
| What percentage of your patients that you suggested topical treatment keep-on receiving the treatment and revisit for follow-up? | 0-20% | 35 | 17.6 |
|  | 21-40% | 45 | 22.6 |
|  | 41-60% | 61 | 30.7 |
|  | 61-80% | 43 | 21.6 |
|  | 81-100% | 15 | 7.5 |
| What is the most common reason of the patients for breaking-up the topical treatment? | Application difficulty | 72 | 36.5 |
|  | Irritation | 121 | 61.4 |
|  | Reactive hyperpigmentation | 24 | 12.2 |
|  | Patient's incompatibility | 137 | 69.5 |
|  | The cost of drugs | 84 | 42.6 |
|  | Difficulty in accessing drugs | 15 | 7.6 |
|  | Patient's preference | 25 | 12.7 |
|  | Ineffectiveness | 47 | 23.9 |
|  | Unable to find an appointment | 1 | 0.5 |
|  | Recurrence | 1 | 0.5 |
| What is your first choice of oral treatment? | Tranexamic acid | 45 | 23.4 |
|  | Polypodium leucotomos extract | 20 | 10.4 |
|  | Glutathione | 5 | 2.6 |
|  | N-Acetyl Cysteine | 1 | 0.5 |
|  | I don’t use oral therapy | 136 | 70.8 |
| Why, if you do not use oral tranexamic acid? | Drug cost | 9 | 5.8 |
|  | Side effect concern | 71 | 46.1 |
|  | No drug experience | 108 | 70.1 |
|  | Difficulty obtaining medication | 28 | 18.2 |
| Do you suggest melasma treatment in summer? | Yes | 65 | 32.7 |
|  | No | 134 | 67.3 |
| Do you have the opportunity of laser and/or light therapy? | Yes | 49 | 24.9 |
|  | No | 148 | 75.1 |
| If you use laser which device do you prefer? | Fractional non-ablative lasers | 7 | 14 |
|  | Fractional ablative lasers | 2 | 4 |
|  | Q-switched Nd-YAG laser | 29 | 58 |
|  | Q-switched ruby laser | 3 | 6 |
|  | Pulse dye laser | 3 | 6 |
|  | Intense pulsed light | 17 | 34 |
|  | Fractional CO_2_ laser | 8 | 16 |
|  | Er-YAG laser | 2 | 4 |
|  | Thulium laser | 3 | 6 |
|  | Fractional Q-switched lasers | 1 | 2 |
|  | Combination | 3 | 6 |
| If you use laser how many sessions do you prefer? | 1 | 1 | 2 |
|  | 2 | 1 | 2 |
|  | 3 | 22 | 44 |
|  | 4 | 4 | 9 |
|  | 5 | 2 | 4 |
|  | 6 | 5 | 10 |
|  | 6< | 10 | 20 |
| What percentage of your patients experience recurrence after melasma treatment? | 0-20 | 7 | 3.9 |
|  | 21-40 | 44 | 24.3 |
|  | 41-60 | 83 | 45.9 |
|  | 61-80 | 38 | 21 |
|  | 81-100 | 9 | 5 |
| After which treatment option do you experience recurrence most? | Topical preparations | 134 | 76.6 |
|  | Oral preparations (Tranexamic acid, Polypodium leucotomos extract etc) | 4 | 2.3 |
|  | Chemical peelings | 9 | 5.1 |
|  | Mesotherapy | 0 | 0 |
|  | Platelet rich plasma | 3 | 1.7 |
|  | Micro needling | 5 | 2.9 |
|  | Radiofrequency micro needling | 0 | 0 |
|  | Dermabrasion | 1 | 0.6 |
|  | Lasers and light therapies | 19 | 10.9 |
| Do you provide maintenance therapy to patients? | Yes | 115 | 59.3 |
|  | No | 79 | 40.7 |
